# Dynamics of osmosis in a porous medium

**DOI:** 10.1098/rsos.140352

**Published:** 2014-11-12

**Authors:** Silvana S. S. Cardoso, Julyan H. E. Cartwright

**Affiliations:** 1 Department of Chemical Engineering and Biotechnology, University of Cambridge, Cambridge CB2 3RA, UK; 2 Instituto Andaluz de Ciencias de la Tierra, CSIC–Universidad de Granada, Campus Fuentenueva, 18071 Granada, Spain

**Keywords:** osmosis, porous medium, semipermeable membrane, Maxwell's demon

## Abstract

We derive from kinetic theory, fluid mechanics and thermodynamics the minimal continuum-level equations governing the flow of a binary, non-electrolytic mixture in an isotropic porous medium with osmotic effects. For dilute mixtures, these equations are linear and in this limit provide a theoretical basis for the widely used semi-empirical relations of Kedem & Katchalsky (Kedem & Katchalsky 1958 *Biochim. Biophys. Acta* **27**, 229–246 (doi:10.1016/0006-3002(58)90330-5), which have hitherto been validated experimentally but not theoretically. The above linearity between the fluxes and the driving forces breaks down for concentrated or non-ideal mixtures, for which our equations go beyond the Kedem–Katchalsky formulation. We show that the heretofore empirical solute permeability coefficient reflects the momentum transfer between the solute molecules that are rejected at a pore entrance and the solvent molecules entering the pore space; it can be related to the inefficiency of a Maxwellian demi-demon.

## 2. Introduction

It seems that there is currently no correct theoretical development of the fundamental equations describing the physics of transport in a porous medium with osmotic effects. At present, all work to model osmotic flow in a porous medium at the continuum level ultimately derives from the semi-empirical 1958 formulation of Kedem & Katchalsky [1]. According to Kedem–Katchalsky, for dilute solutions, the molar flux of solute (species 1), *N*_1_, and volume flux of solvent (species 2), *u*_2_, across a membrane permeable to the solvent but only partially permeable to the solute, are

2.1 $$\left.\begin{array}{rl} u_{2} & =L(-\delta p+\sigma RT\delta c_{1})\\ \text{and}\;N_{1} & =-wRT\delta c_{1}+(1-\sigma )u_{2}c_{1}. \end{array}\right\}$$

These relations were obtained from non-equilibrium thermodynamics under the assumption of linearity between the fluxes and the driving forces. Here *R* is the universal gas constant and the temperature *T* is assumed constant; *δp* and *δc*_1_ are the pressure and molar concentration differences across the membrane, and *L* is a transport coefficient. The reflection coefficient *σ* measures the fraction of solute molecules that are reflected by the membrane [2], taking the value of one for a perfectly semipermeable membrane and zero for a completely permeable one. The solute permeability coefficient *w* is null for a semipermeable membrane (*σ*=1); little is known about the physical meaning of *w*. The phenomenological coefficients *w*, *σ* and *L* are measured, for a given solute and membrane, by carefully designed experiments [3]. Post Kedem and Katchalsky, earlier theoretical proposals for the interaction of osmosis and viscous flow in a porous medium include the use of a potential-energy field [4] and of friction factors [5], but neither of these approaches relate the ad-hoc coefficients introduced therein to the properties of the solution and of the porous medium. Later work relied on a dusty-gas type model [6,7], but has been proved erroneous [8] owing to a double count of the viscous forces in the fluid and at the fluid–solid boundaries; this model also emphasized the significance of a ‘partial osmotic pressure’ but failure to distinguish between equilibrium static and non-equilibrium flow situations may have caused confusion. A similar error has propagated into subsequent literature [9]. More recent models [10,11] have taken into account electrostatic effects outside the pore entrance and exit to describe the osmotic pressure in terms of an electric double-layer potential; however, these works do not apply to a non-electrolytic system. In this work, we perform a momentum balance at the molecular level to derive the minimal continuum-level equations for the flow of a binary, non-electrolytic mixture in a porous medium in the presence of osmotic effects. We discuss the conditions under which these equations may be reduced to the simplified, semi-empirical form above, and we address the physical significance of the solute permeability coefficient, *w*.

The classical treatment of osmosis considers a system in thermodynamic equilibrium. While the early works of van't Hoff [12] and Rayleigh [13] explained osmosis in terms of the work done by the rebounding molecules of solute on a selective (semipermeable) membrane, the same phenomenon was later described by Gibbs onwards in terms of the free energy and chemical potential [14]. Different disciplines have preferred one or the other of these approaches to derive the classical thermostatic result, but the kinetic and thermodynamic theoretical treatments are entirely equivalent [15]. However, to quantify the evolution of a system towards such equilibrium, the flux laws governing the flows of solute and solvent are necessary. Recent osmotic research has focused on molecular-dynamics simulations of flow, for example in nanopores [16], in nanotube arrays [17] and in nanofluidic diodes [18]. But, in spite of such numerical studies, little is known about the key intermolecular and molecule–pore interactions that drive osmotic flow in a pore and how these relate to continuum-level properties of the fluid and the porous matrix. Yet, it is this translation of the molecular behaviour to a mesoscale involving many pore lengths, connecting the atomic scale and the macroscopic scale, that is of utmost importance for the understanding of the role of osmosis in all its manifold applications in physics, chemistry and biology. Thus, we follow in the spirit of Einstein's study of Brownian motion [19] in coupling a kinematic approach to osmosis with fluid mechanics.

In order to develop a simple theoretical argument, we focus on core mechanisms and make the following assumptions. (i) The porous medium is rigid, isotropic and homogeneous. (ii) The porous medium and the fluid are in thermal equilibrium and isothermal. (iii) The mixture is binary and non-electrolytic. (iv) The solute is inviscid and the solvent is viscous. The introduction of solute–solute interactions through a viscosity for the solute is straightforward [8], but complicates the mathematical presentation. We have opted to keep the model as simple as possible. (v) The interactions between solute and solvent molecules are represented by the Maxwell–Stefan diffusivity of the solute in a binary mixture of solute and the solvent. A discussion of this assumption is provided in the text. (vi) We neglect possible chemical reaction of the species with each other and solvation at the pore wall. These effects may be introduced in a further development of the model [9]. (vii) We assume the flow has low Reynolds and Péclet numbers. The validity of this assumption is discussed in the text.

## 3. Theoretical derivation

**Figure 1.**
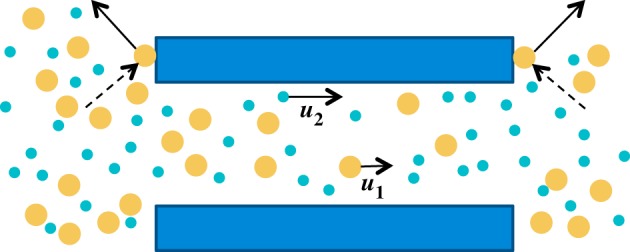
Flow of solute and solvent molecules near a pore entrance and exit. Some of the solute molecules rebound from the pore entrance and subsequently transfer part of their momentum to neighbouring solvent molecules through collisions. A similar process happens at the pore exit. A difference in the concentrations of solute between the entrance and exit creates an osmotic force. (The instantaneous velocity of a single solute molecule impacting at the pore boundary is much larger than the solute average velocity shown inside the pore; the arrows are not drawn to scale.)

Consider the isothermal flow of a binary fluid, comprising a non-electrolytic solute species 1 and a solvent species 2, in a porous medium (figure 1). The medium is completely permeable to the molecules of a viscous solvent but only partially permeable to an inviscid solute owing to chemical or physical effects. Thus, as the solution flows in a given direction within the pore space, a fraction *σ* of the molecules of solute are reflected backward after elastic collision with the solid wall at the entrance to the pore; the remainder flow forward into the pore space, where they undergo further elastic collisions. The molecules of solvent do not rebound upon striking the solid walls, but stick to the wall and later leave it with zero average velocity parallel to the pore wall [20]. The mass and energy fluxes of incident and emitted molecules are equal. However, momentum is not equal for the fluxes of incident and released molecules at the wall; indeed, viscous shear will transfer momentum to the pore wall. We next quantify each of the momentum changes undergone by the solute and the solvent molecules. As the molecules of solute move through the pores of the solid matrix, they change momentum owing to two different types of interactions: collisions with molecules of solvent within the pore and collisions with the walls of the solid matrix. The rate of change of momentum of molecules of species 1, per unit volume of mixture in a pure fluid medium, resulting from collision with molecules of species 2 is *RTc*_1_*c*_2_(***u***_**1**_−***u***_**2**_)/((*c*_1_+*c*_2_)*D*_12_) [21]. In elementary kinetic theory of diffusion [20], the product of concentrations of solute and solvent *c*_1_*c*_2_ reflects the number of collisions and the difference in the average velocities (***u***_**1**_−***u***_**2**_), the average momentum exchanged in a single elastic collision of smooth, rigid, spherical molecules. Of course, in reality more complex effects may arise through non-elastic collisions, possible multiple molecular encounters, the effect of non-uniformities in composition and pressure on the Maxwellian velocity distribution of the molecules, and the presence of internal as well as translational molecular energy [20]. However, the physical interpretation of the Maxwell–Stefan diffusivity of the solute in the binary mixture of solute and solvent, *D*_12_, as an inverse drag coefficient remains valid [21], whether the frictional drag exerted by one set of molecules moving through the other arises purely from binary elastic intermolecular collisions or from more complex interactions. In the porous medium, only a fraction (1−*σ*) of solute molecules enters a pore, so that the number of collisions is proportionally reduced. Also, the molecules move in tortuous paths around the solid, so that the flux of momentum in any particular direction is reduced by a factor $1/\tau =\bar{\cos^{2}\theta }$, where *θ* is the inclination of a pore relative to the direction specified and the bar represents an average over all pore directions; *τ* is the tortuosity of the porous matrix [22]. The rate of change of momentum of molecules of species 1, per unit volume of mixture in a porous medium, resulting from collisions with molecules of species 2 is then

3.1 $$RT(1-\sigma )\frac{c_{1}c_{2}\tau ({\text{u}}_{\text{2}}-{\text{u}}_{\text{1}})}{(c_{1}+c_{2})D_{12}}.$$

The fraction *σ* of the molecules of solute that impact on the solid and rebound at the entrance and exit of a pore undergo a change in momentum, per unit volume of fluid, of magnitude

3.2 $$\frac{1}{2}\sigma RT\nabla c_{1}-(-\frac{1}{2}\sigma RT\nabla c_{1})=\sigma RT\nabla c_{1},$$

where *σRT***∇***c*/2 is the momentum of molecules leaving the solid surface and −*σRT***∇***c*/2 is the momentum of the molecules impacting on the solid. This can be viewed as a gradient of osmotic pressure, and *σ* can be related to the ratio of the solute molecule size and the pore size; the solvent molecules are regarded as essentially infinitesimal in size. After the solute molecules rebound from the porous medium, a fraction of their momentum is transferred to neighbouring solvent molecules through collisions. This fraction of momentum depends on the distribution of solute and solvent molecules near the rebounding surface, which is unknown; we assume it takes a constant average value of 2*β*. Thus, the change in momentum of the solute molecules caused by collision with the solid surface and with neighbouring solvent molecules, per unit volume of fluid, is

3.3 $$\frac{1}{2}\sigma RT\nabla c_{1}+(1-2\beta )\frac{1}{2}\sigma RT\nabla c_{1}=(1-\beta )\sigma RT\nabla c_{1}.$$

To the best of the authors' knowledge, the introduction of the parameter *β* representing the exchange of momentum through collision between rebounding solute molecules and solvent molecules near a pore entrance and exit is novel. From a physical point of view, it is clear that such transfer takes place, but how important is it? We shall confirm later that *β* is indeed non-zero, and can be related to the solute permeability coefficient *w* used in the phenomenological model of Kedem & Katchalsky [1] and more recently measured experimentally [3].

The total change in momentum of the molecules of solute is balanced by the driving force from the gradient in chemical potential of the solute, *g*_1_, expressed per unit volume of fluid as [21]

3.4 $$c_{1}\nabla_{T}g_{1}=c_{1}\nabla_{T,p}g_{1}+\phi_{1}\nabla p,$$

where *ϕ*_1_ is the volume fraction of the solute in the mixture. In effect, this is the driving force for entropy production owing to irreversible processes [14,23,24]. The momentum balance for the solute may thus be written as follows:

3.5 $$c_{1}\nabla_{T,p}g_{1}+\phi_{1}\nabla p=RT(1-\sigma )\frac{c_{1}c_{2}\tau ({\text{u}}_{\text{2}}-{\text{u}}_{\text{1}})}{(c_{1}+c_{2})D_{12}}+(1-\beta )\sigma RT\nabla c_{1}.$$

Each of the quantities on the right-hand side of this equation arise from terms (3.1) and (3.3) discussed earlier. A momentum balance for the molecules of solvent leads to a similar equation

3.6 $$c_{2}\nabla_{T,p}g_{2}+\phi_{2}\nabla p=RT(1-\sigma )\frac{c_{1}c_{2}\tau ({\text{u}}_{\text{1}}-{\text{u}}_{\text{2}})}{(c_{1}+c_{2})D_{12}}+\beta \sigma RT\nabla c_{1}-\frac{\mu_{2}}{k_{2}}\phi_{2}{\text{u}}_{\text{2}}.$$

Here *μ*_2_ is the viscosity and *ϕ*_2_ the volume fraction of the solvent in the mixture; in general, the permeability of the medium to the solvent in the presence of the solute, *k*_2_, varies with the composition of the mixture. The first term on the right-hand side accounts for the momentum change of the solvent molecules upon collision with the solute molecules in the pore. The second term quantifies the momentum change of the solvent molecules through collision with rebounding solute molecules at the entrance and exit of the pore; as mentioned above, a fraction 2*β* of the momentum of the rebounding solute molecules is transferred to the solvent. The last term accounts for the loss of momentum of the solvent molecules upon collision and sticking at the solid surface inside the pore, as described earlier. It quantifies the effect of viscous forces averaged over many pore orientations and gives rise to Darcy's law for flow in a porous medium [22].

In the momentum balances above for the solute and solvent, we have assumed that the mean free path of the molecules is sufficiently smaller than the pore diameter in the solid matrix, so that the fluid may be modelled as a continuum with constant transport properties such as viscosity and diffusivity. We have also assumed that the momentum change arising from the acceleration or deceleration of the fluid as it moves through the tortuous paths in the porous medium is negligible; this assumption is valid [22] for low Reynolds number flows such that *Re*_2_=*ρ*_2_|***u***_**2**_|*δ*/*μ*_2_≪1, where *ρ*_2_ is the density of the solvent and *δ* is the typical pore length scale. The effects of dispersion of a component arising from such tortuous motion have been neglected, which is acceptable when the Péclet number of the flow is small, *Pe*_*i*_=|***u***_***i***_|*δ*/*D*_12_≪1 (*i*=1,2) [22].

The sum of equations (3.5) and (3.6) quantifies the pressure gradient in terms of the velocity of the solvent and the osmotic effect of the solute

3.7 $$\nabla p=-\frac{\mu_{2}}{k_{2}}\phi_{2}{\text{u}}_{\text{2}}+\sigma RT\nabla c_{1}.$$

Substituting equation (3.7) into (3.5) leads to a relationship between the velocities of the solute and solvent,

3.8 $$\left[RT(1-\sigma )\frac{c_{1}c_{2}\tau }{(c_{1}+c_{2})D_{12}}\right]({\text{u}}_{\text{1}}-{\text{u}}_{\text{2}})+\phi_{1}\phi_{2}\frac{\mu_{2}}{k_{2}}{\text{u}}_{\text{2}}=-RT[\Gamma_{1}-(\phi_{2}-\beta )\sigma ]\nabla c_{1},$$

where we have used *c*_*i*_**∇**_*T*,*p*_*g*_*i*_=*RTΓ*_*i*_**∇***c*_*i*_ with *Γ*_*i*_=(*c*_1_+*c*_2_−*c*_*i*_)/[(*c*_1_+*c*_2_)(1−*ϕ*_*i*_)], valid for an ideal solution; for non-ideal behaviour one may introduce activity coefficients [23] in a straightforward manner. Solving equations (3.7) and (3.8) for the velocities of the solute and solvent leads to

3.9 $$\left.\begin{array}{rl} {\text{u}}_{\text{1}} & =\frac{(c_{1}+c_{2})D_{12}}{\tau c_{1}c_{2}}\left[\frac{\phi_{1}}{RT(1-\sigma )}\nabla p-\frac{\Gamma -(\phi_{2}-\phi_{1}-\beta )\sigma }{1-\sigma }\nabla c_{1}\right]+{\text{u}}_{\text{2}}\\ \text{and}\;{\text{u}}_{\text{2}} & =\frac{k_{2}}{\mu_{2}\phi_{2}}(-\nabla p+\sigma RT\nabla c_{1}). \end{array}\right\}$$

The volumetric flux of solvent and the molar flux of solute, per unit area of porous medium, are, respectively, given by

3.10 $$\left.\begin{array}{rl} \text{u} & =\varepsilon {\text{u}}_{\text{2}}=\frac{k_{2}\varepsilon }{\mu_{2}\phi_{2}}\left(-\nabla p+\sigma RT\nabla c_{1}\right)\\ \text{and}\;{\text{N}}_{\text{1}} & =(1-\sigma )c_{1}\varepsilon {\text{u}}_{\text{1}}=\frac{c_{1}+c_{2}}{c_{2}}\frac{\varepsilon D_{12}}{\tau }\\ & \;\times \left(\frac{\phi_{1}}{RT}\nabla p-[\Gamma -(\phi_{2}-\phi_{1}-\beta )\sigma ]\nabla c_{1}\right)+(1-\sigma )c_{1}\text{u}, \end{array}\right\}$$

where *ε* is the porosity of the medium. We recognize in *εD*_12_/*τ* the effective diffusivity of the solute in the solvent in the porous medium. For a dilute solution (i.e. in the limit $\phi_{1}\to 0$, $\phi_{2}\to 1$), equations (3.9) simplify to

3.11 $$\left.\begin{array}{rl} {\text{u}}_{\text{1}} & =-\frac{1-(1-\beta )\sigma }{1-\sigma }\frac{D_{12}}{\tau c_{1}}\nabla c_{1}+{\text{u}}_{\text{2}}\\ \text{and}\;{\text{u}}_{\text{2}} & =\frac{k_{2}}{\mu_{2}}(-\nabla p+\sigma RT\nabla c_{1}). \end{array}\right\}$$

These relations show that the slip velocity between the solute and the solvent arises essentially from the transfer of momentum from interspecies molecular collisions, i.e. frictional drag between the species, but not from the presence of the solid matrix. The flow of solvent is affected by viscous stresses between the fluid and the solid matrix, and the gradient in osmotic pressure owing to the solute. For a dilute solution, the volumetric flux of solvent and the molar flux of solute, per unit area of porous medium, are, respectively, given by

3.12 $$\left.\begin{array}{rl} \text{u} & =\varepsilon {\text{u}}_{\text{2}}=\frac{k_{2}\varepsilon }{\mu_{2}}(-\nabla p+\sigma RT\nabla c_{1})\\ \text{and}\;{\text{N}}_{\text{1}} & =(1-\sigma )c_{1}\varepsilon {\text{u}}_{\text{1}}=-[1-(1-\beta )\sigma ]\frac{\varepsilon D_{12}}{\tau }\nabla c_{1}+(1-\sigma )c_{1}\text{u}. \end{array}\right\}$$

Equations (3.12) describe the flow of an ideal dilute binary mixture in an isotropic porous medium, with osmotic effects arising from the interaction of the solute molecules with the solid matrix. The derivation presented here may be easily extended, for instance, to a multi-component mixture with non-ideal behaviour, and to include the gravitational force.

## 4. Discussion

Equations (3.12) have the structure of the semi-empirical equations proposed by Kedem & Katchalsky [1] given in equations (2.1). The coefficients are expressed in terms of the properties of the solute, the solvent and the porous matrix, and satisfy Onsager's reciprocal relation [25] in that (∂***u***/∂*c*_1_)_*p*_=*RT*[∂(***N***_**1**_/*c*_1_−***u***)/∂*p*]_*c*_1__. The present work shows that linearity between the fluxes and the driving forces holds for dilute, ideal mixtures, but not for more concentrated or non-ideal ones, for which the slip velocity between the solvent and solute is complex (see equation (3.8)). The solute permeability *w* reflects the momentum of the solute molecules after rebounding near a pore entrance and exit. It thus represents the efficiency of the sorting process being carried out, and so we might see the coefficient *β* as the inefficiency of the particular Maxwellian demi-demon of the pore (a complete Maxwell demon [26,27] would require two semipermeable membranes back to back, as Szilard discussed [28,29]).

We hope that this derivation will be of utility to the many people who use the Kedem–Katchalsky equations, which have hitherto been validated experimentally but not theoretically. The present work moreover goes beyond Kedem and Katchalsky to the nonlinear regime of concentrated or non-ideal mixtures. We anticipate that it will stimulate future molecular-dynamical simulations to explore the role of interspecies momentum transfer at the entrance and exit of nanopores on osmosis, and their impact on the continuum-level behaviour of the fluid in a porous medium.
